# Supplementary figures and images for: Native to designed: microbial -amylases for industrial applications
Source: PeerJ. 2021 May 18;9:e11315. doi: 10.7717/peerj.11315 (PMC8139272; doi:10.7717/peerj.11315)

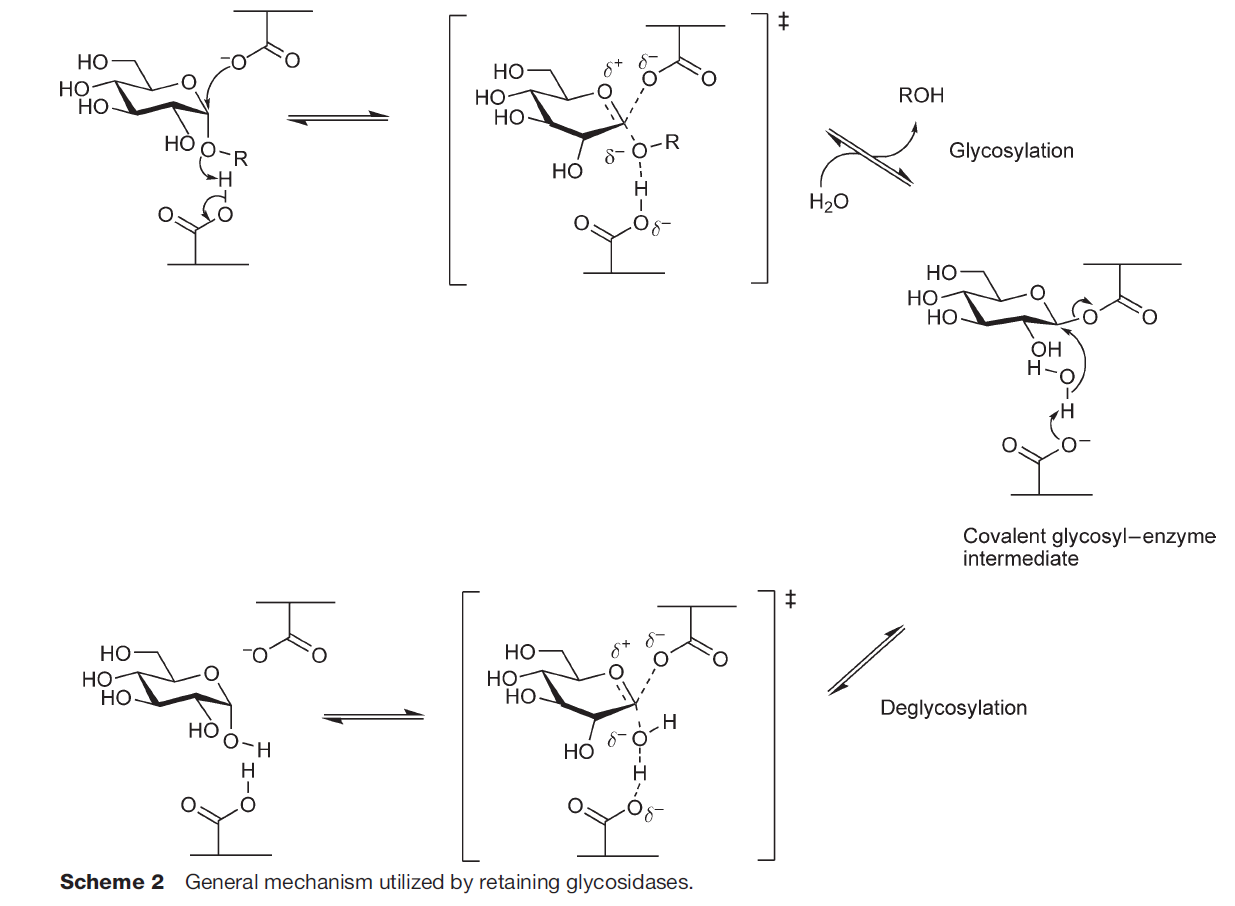

Supplement: Supplemental Information 1 [file peerj-09-11315-s001.png]

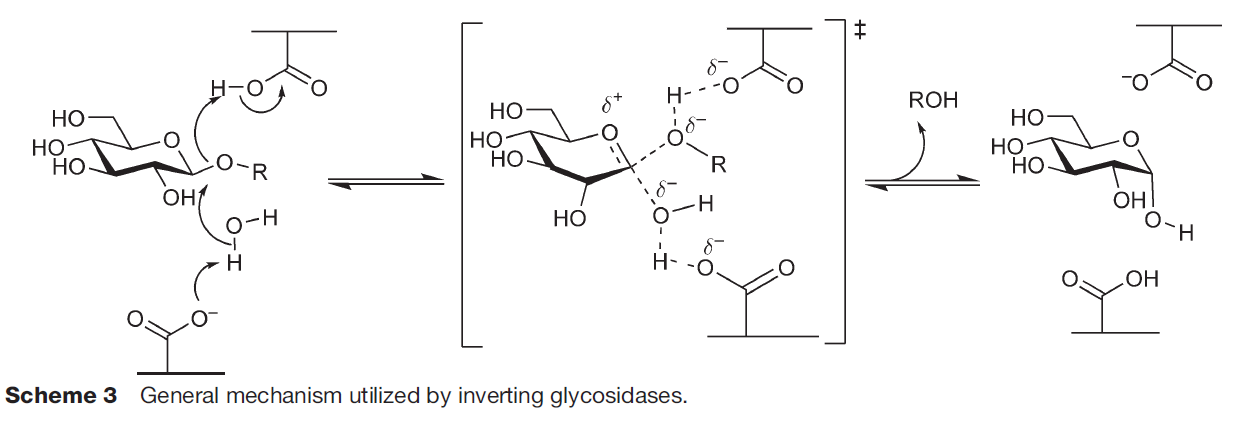

Supplement: Supplemental Information 2 [file peerj-09-11315-s002.png]
